# Molecular detection and phylogenetic analyses of *Wolbachia* in natural populations of nine galling Aphid species

**DOI:** 10.1038/s41598-020-68925-z

**Published:** 2020-07-21

**Authors:** Weibin Ren, Hongyuan Wei, Ying Yang, Shuxia Shao, Haixia Wu, Xiaoming Chen, Zixiang Yang

**Affiliations:** 1 Research Institute of Resource Insects, Chinese Academy of Forestry, Kunming, China; 2 Key Laboratory of Cultivating and Utilization of Resources Insects, State Forestry Administration, Kunming, China

**Keywords:** Bacteria, Pathogens

## Abstract

*Wolbachia* is one of the most abundant facultative intracellular symbionts in arthropods. It alters host biology in diverse ways, including the induction of reproductive manipulation, association of nutrient supplier and protection against pathogens. Aphids are a group of insects which exhibit interesting biological characteristics such as complex life cycles, alteration of sexual and asexual reproduction and shifts between two different hosts. *Wolbachia* is widely present in many orders of insects, but so far limited studies on *Wolbachia* in aphids have been carried out. Galling aphids are a group of aphids that induce galls on their primary host plants at specific life stage. In this study, 15 natural populations representing nine galling aphid species were analyzed for the presence of *Wolbachia* using species-specific primer pairs. *Wolbachia* presence in galling aphids was quite low and varied significantly among aphid populations. Only three of the 15 populations we analyzed had detectable *Wolbachia* and the overall infection rate was 20%. Two *Wolbachia* strains, O and B, were identified from the galling aphids *Kaburagia rhusicola* and *Schlechtendalia chinensis*. Strain O was for the first time to be found in aphids, and it is likely involved with the life stages of galling aphids living in closed microenvironments with specific survival strategies that are different from free-living aphids.

## Introduction

*Wolbachia* is an intracellular facultative symbiont present widely in arthropods. *Wolbachia* species such as *Wolbachia pipientis* have many different strains^1,2^. At present, 16 *Wolbachia* supergroups have been reported, and named A to F and H to Q from insects^3–6^. *Wolbachia* diversity was initially characterized using the genes *wsp*, *16S rRNA*, *ftsZ*, *gltA*, and *groEL* as molecular markers^7^. One of the consequences of *Wolbachia* in insects is associated with the induction of different reproductive strategies such as parthenogenesis, feminization, male-killing and cytoplasmic incompatibility (CI)^8–11^. In addition to reproduction, *Wolbachia* can also affect the biological characteristics of its hosts, such as providing host nutrients, protecting hosts from RNA viruses, regulating the age structure of the host populations, and improving the proliferation of host stem cells^13–16^. The presence of *Wolbachia* in insects may put selective pressure more strongly on a host or create reproductive barriers that can lead to speciation. *Wolbachia*-based methods have also been developed to control the transmission of insect pests and arboviruses^17,18^. Aphids are a group of insects, many of which are important pests for agriculture and forestry^18,19^. However, some species such as galling aphids can manipulate to plant tissues, resulting in the formation of galls, which can provide protection for aphids from predators^20–22^. Like most aphids, galling aphids exhibit complex biological traits and a complicated life cycle, such as sexual and asexual reproduction, and alternating between two host species^23^. Among galling aphids, Chinese galling aphids are specific species which induce closed galls rich in tannins on Chinese sumac trees (Anacardiaceae: *Rhus*) and have been used for medicinal, chemical and other industrial purposes^23,24^.

Until recently, aphids were thought to be free of *Wolbachia*^26–29^. However, data on European aphids indicate that the absence of *Wolbachia* was an underestimate^3^. A total of 425 natural samples representing 144 aphid species have been analyzed for the presence of *Wolbachia* using specific *16S rRNA*-based PCR, and 37 (8.7%) samples have been found to have *Wolbachia*^3^. Later in China, 109 samples representing 73 aphid species have also been analyzed, and all the samples have been found to have *Wolbachia*^4^. Wang et al. (2014)^2^ suggested that the infection status of *Wolbachia* in aphid populations from China might differ from other areas and this multiple infection pattern was probably caused by horizontal transmission^2,30,31^. However, horizontal transmission has not been observed in European aphids.

So far, four *Wolbachia* supergroups, A, B, M and N have been detected in aphids with supergroup M being the most commonly detected^3^. The distribution patterns of *Wolbachia* in Chinese aphids are complex and varied among different species^4^. *Wolbachia* in aphids is underestimated primarily because of its low titer and/or high divergence of different strains. Among the 217 aphid species analyzed for *Wolbachia* before, only 11 were galling aphids (4.1%), and of those, only six galling aphids carried *Wolbachia*^3,4^. Therefore, the distribution of *Wolbachia* in galling aphids and its significance remain to be studied.

The objective of this study is to investigate the presence of *Wolbachia* in natural populations of galling aphids from China. Specifically, *Wolbachia* strains were screened and classified based on nine marker genes: *16S rRNA*, *gltA*, *groEL*, *wsp, gatB*, *fbpA*, *coxA*, *hcpA* and *ftsZ*. Phylogenetic analysis was also conducted based on the sequences of the marker genes.

## Results

### Screening for *Wolbachia* in natural populations of galling Aphids

A total of 117 samples of natural galling aphids were collected from various host plants in 7 different locations in China. The 117 samples represented 15 populations of 9 aphid species within 6 genera, 2 tribes of Aphididae. The 15 populations were from genera *Kaburagia* (1 species, 5 populations), *Floraphis* (1 species, 1 populations), *Schlechtendalia* (2 species, 4 populations), *Nurudea* (1 species, 1 populations), *Pemphigus* (3 species, 3 populations), and *Chaetogeoica* (1 species, 1 populations) (Table 1). All samples were screened for the presence of *Wolbachia* by PCR amplification using *16S rRNA*-specific primers 16S-281F/1372R. Our results showed that the presence of *Wolbachia* in these populations was infrequent and varied significantly among different aphid populations. Among all 15 aphid populations, only 3 populations were detected to harbor *Wolbachia*. These *Wolbachia*-carrying aphids were *Kaburagia rhusicola* and *Schlechtendalia chinensis* from the tribe Fordini, and *Pemphigus yunnanensis* from the tribe Pemphigini. No *Wolbachia* was detected in the remaining populations. Among the *Wolbachia-*positive species, *Wolbachia* was detected in four out of six samples in *K. rhusicola*, and in one out of nine samples in either *S. chinensis* or *P. yunnanensis* (Table 1).

**Table 1:** Sample for the study of *Wolbachia* infection in galling aphid.

Aphid species	Primary host	Collected location	*Wolbachia* infection prevalence										Number of tested populations	Number of infected populations
			*16S rRNA*	MLST genes					Other genes			Supergroup		
				*gatB*	*fbpA*	*coxA*	*hcpA*	*ftsZ*	*wsp*	*gltA*	*groEL*			
*Kaburagia rhusicola*	*Rhus potaninii*	Yunnan Kunming	+	+	+	−	−	−	−	−	−	O	6	4
		Shaanxi Chenggu	−	−	−	−	−	−	−	−	−	−	6	0
		Shaanxi Ningqiang	−	−	−	−	−	−	−	−	−	−	6	0
		Yunman Yanjin	−	−	−	−	−	−	−	−	−	−	6	0
		Sichuan Emei	−	−	−	−	−	−	−	−	−	−	6	0
*Floraphis meitanensis*	*Rhus punjabensis* var. *sinica*	Yunnan Kunming	−	−	−	−	−	−	−	−	−	−	9	0
*Schlechtendalia peitan*	*Rhus chinensis*	Yunnan Kunming	−	−	−	−	−	−	−	−	−	−	9	0
*Nurudea shiraii*	*Rhus chinensis*	Yunnan Kunming	−	−	−	−	−	−	−	−	−	−	9	0
*Schlechtendalia chinensis*	*Rhus chinensis*	Yunnan Kunming	−	−	−	−	−	−	−	−	−	−	9	0
		Hubei Wufeng	−	−	−	−	−	−	−	−	−	−	9	0
		Sichuan Emei	+	+	+	−	−	−	−	−	−	O	9	1
*Pemphigus yangcola*	*Populus yunnanensis*	Yunnan Kunming	−	−	−	−	−	−	−	−	−	−	6	0
*Pemphigus yunnanensis*	*Populus yunnanensis*	Yunnan Kunming	+	+	+	+	+	+	−	−	−	B	9	1
*Pemphigus populitransversus*	*Populus yunnanensis*	Yunnan Kunming	−	−	−	−	−	−	−	−	−	−	9	0
*Chaetogeoica folidentata*	*Pistacia chinensis*	Yunnan Chuxiong	−	−	−	−	−	−	−	−	−	−	9	0

+, amplification; −, failed to detect amplification product.

### Sequence variation in *Wolbachia* genes

Nine genes including *16S rRNA*, *gltA*, *groEL*, *wsp*, *gatB*, *fbpA*, *coxA*, *hcpA* and *ftsZ* of *Wolbachia* were selected for PCR amplification. However, only six genes were amplified from at least one of the three populations harboring *Wolbachia*. Three genes including *16S rRNA*, *gatB* and *fbpA* were amplified from *K. rhusicola* and *S. chinensis*. The amplicon lengths were 1,077 bp for *16S rRNA*, 497 bp for *gatB*, and 476 bp for *fbpA* for samples from *K. rhusicola*, whereas the amplicons were 1,061 bp for *16S rRNA*, 492 bp for *gatB*, and 479 bp for *fbpA* for samples from the *S. chinensis*. Six genes including *16S rRNA*, *gatB*, *fbpA*, *coxA*, *hcpA* and *ftsZ* were amplified from the *P. yunnanensis*. The amplicon sizes were 1,054 bp for *16S rRNA*, 497 bp for *gatB*, 480 bp for *fbpA*, 488 bp for *coxA*, 509 bp for *hcpA*, and 518 bp for *ftsZ* (Table 2)*.*

**Table 2:** Gene sequence lengths of *Wolbachia* infected in the galling aphids.

Aphids and collected location	Gene names					
	*16S rRNA*	MLST genes				
		*gatB*	*fbpA*	*coxA*	*hcpA*	*ftsZ*
*Kaburagia rhusicola* Kunming	1,077	497	476	–	–	–
*Schlechtendalia chinensis* Emei	1,061	492	479	–	–	–
*Pemphigus* yunnanensis Kunming	1,054	497	480	488	509	518

### Phylogenetic analysis

**Figure 1:**
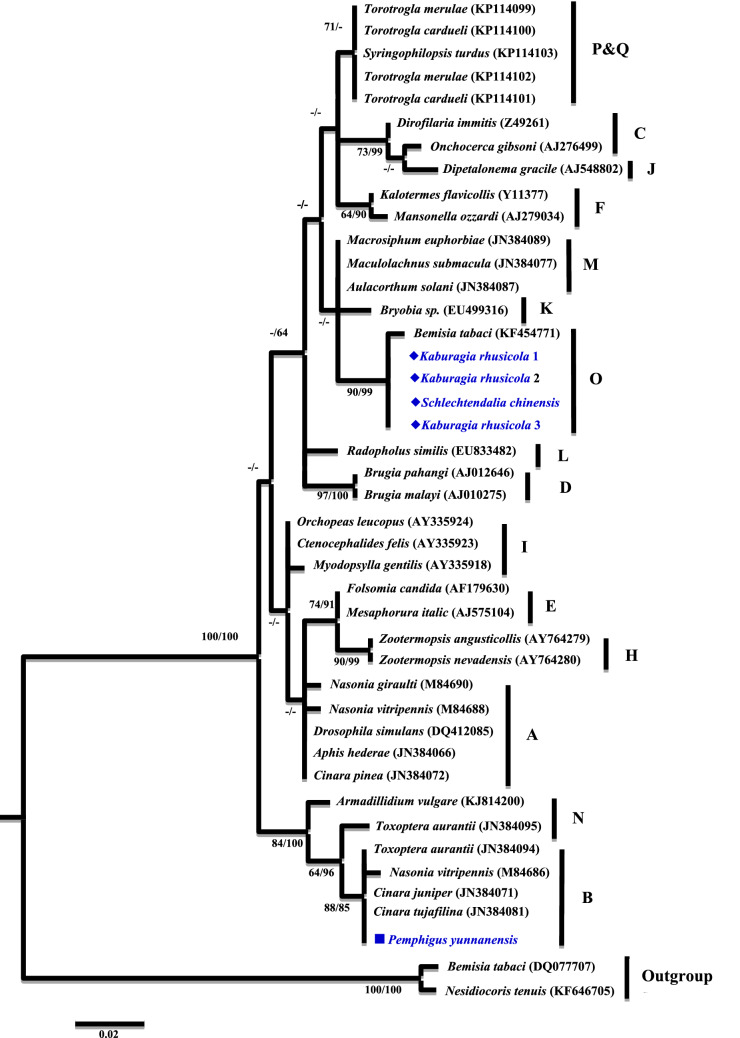
Phylogenetic analysis inferred from *Wolbachia 16S rRNA* gene sequences using Maximum Likelihood (ML) and Bayesian Inference (BI). Scale bar indicates substitutions per site. Aphid *K. rhusicola* and *S. chinensis* indicated by ‘filled diamonds’, and *P. yunnanensis* indicated by ‘filled squares’. ‘–’ indicated support rate less than 50%.

**Figure 2:**
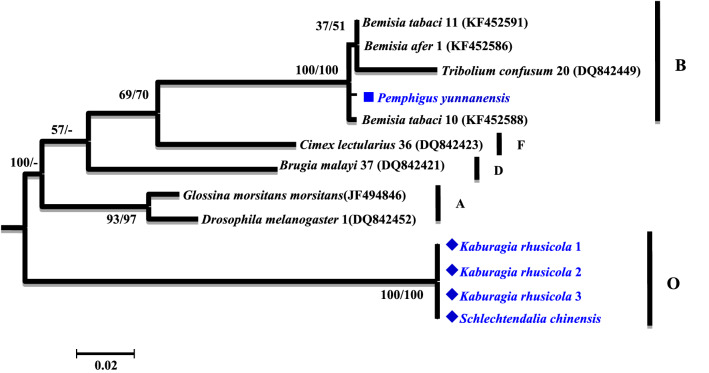
Phylogenetic analysis inferred from *Wolbachia gatB* gene sequences using Maximum Likelihood (ML) and Bayesian Inference (BI). Scale bar indicates substitutions per site. Aphid *K. rhusicola* and *S. chinensis* indicated by ‘filled diamonds’, and *P. yunnanensis* indicated by ‘filled squares’. ‘–’ indicated support rate less than 50%.

**Figure 3:**
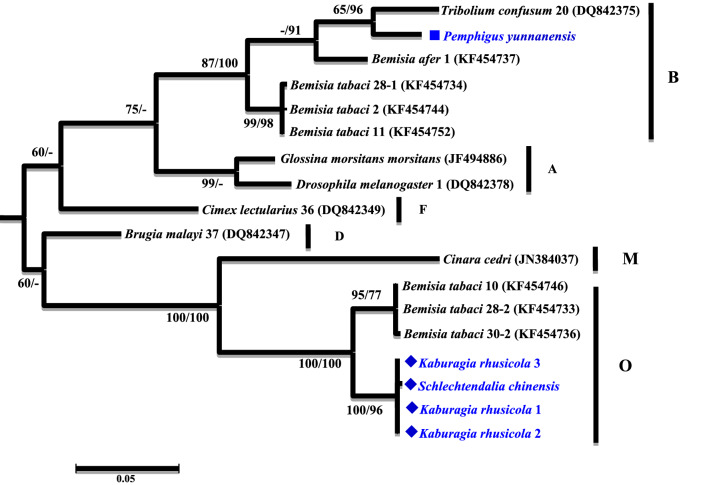
Phylogenetic analysis inferred from *Wolbachia fbpA* gene sequences using Maximum Likelihood (ML) and Bayesian Inference (BI). Scale bar indicates substitutions per site. Aphid *K. rhusicola* and *S. chinensis* indicated by ‘filled diamonds’, and *P. yunnanensis* indicated by ‘filled squares’. ‘–’ indicated support rate less than 50%.

**Figure 4:**
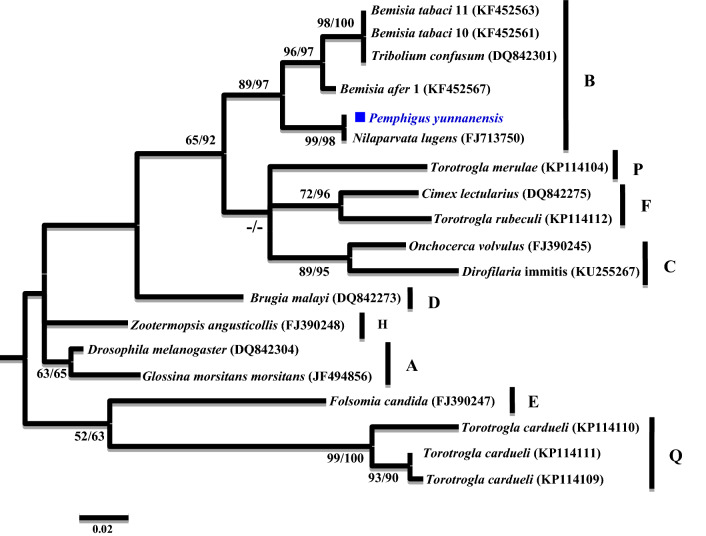
Phylogenetic analysis inferred from *Wolbachia coxA* gene sequences using Maximum Likelihood (ML) and Bayesian Inference (BI). Scale bar indicates substitutions per site. Aphid *P. yunnanensis* indicated by ‘filled squares’. ‘–’ indicated support rate less than 50%.

**Figure 5:**
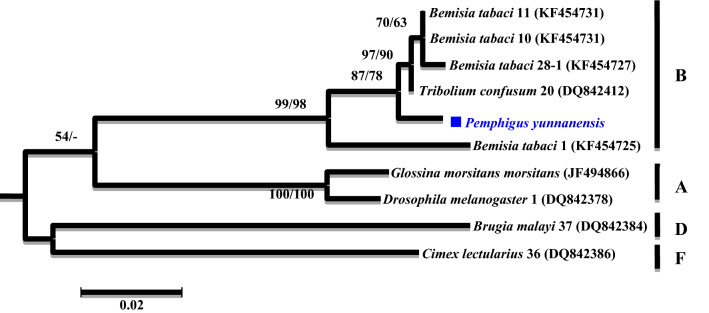
Phylogenetic analysis inferred from *Wolbachia hcpA* gene sequences using Maximum Likelihood (ML) and Bayesian Inference (BI). Scale bar indicates substitutions per site. Aphid *P. yunnanensis* indicated by ‘filled squares’. ‘–’ indicated support rate less than 50%.

**Figure 6:**
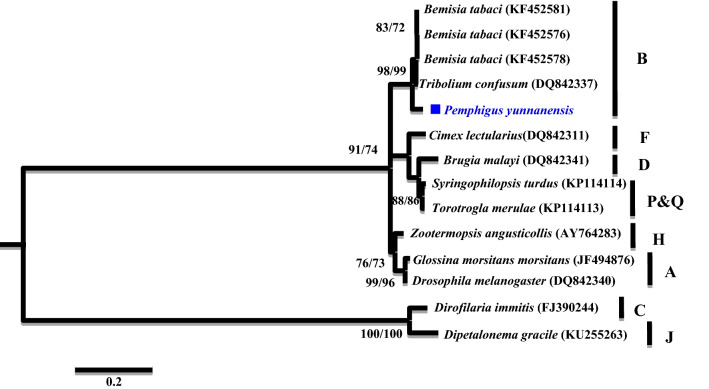
Phylogenetic analysis inferred from *Wolbachia ftsZ* gene sequences using Maximum Likelihood (ML) and Bayesian Inference (BI). Scale bar indicates substitutions per site. Aphid *P. yunnanensis* indicated by ‘filled squares’. ‘–’ indicated support rate less than 50%.

A phylogenetic analysis was conducted based on the partial *16S rRNA* gene sequences (about 1,100 bp). Two different groups were revealed. The *Wolbachia* strains detected from *K. rhusicola* and *S. chinensis* clustered within supergroup O with high bootstrap support (90/99), while strains detected from the *P. yunnanensis* clustered with supergroup B with high bootstrap support values (88/85) (Fig. 1). Moreover, *Wolbachia* strains detected from *K. rhusicola* and *S. chinensis* formed distinct *Wolbachia* lineage analogous to *Wolbachia* supergroup O based on *gatB* and *fbpA* sequences with high bootstrap (100/100, 100/96) (Figs. 2, 3). *Wolbachia* strains detected from *P. yunnanensis* clustered within supergroup B with moderate bootstrap support (88/85) based on partial *16S rRNA* gene sequences, and on *coxA*, *hcpA* and *ftsZ* gene sequences with high or moderate bootstrap (99/98, 87/78, 98/99) (Figs. 4, 5, 6).

## Discussion

There are 16 supergroups of *Wolbachia* that have been identified in insects at present^3–5^. Four of them, named supergroup A, B, M and N, have been detected in aphids. So far *Wolbachia* was detected mostly in free-living aphids^3,4^. In our study, supergroup O and B were detected from three galling aphid species, including *K. rhusicola*, *S. chinensis* and *P. yunnanensis*. Supergroup O has not been reported from aphids before our study. Supergroup O from these two galling aphids was clustered with the supergroup O from a whitefly, forming a distinct *Wolbachia* lineage, which is analogous to the linkage formed based on *gatB* and *fbpA* gene sequences with robust bootstrap values (Figs. 1, 2, 3).

The relation between aphids and their facultative symbiont *Wolbachia* can be affected by different factors such as the ability of symbionts to spread from aphids to aphids within or across populations, the cost of infection for hosts, and aphid living environments (host plant, natural enemy pressure, or temperature)^32,33^. Compared to non-galling aphids, most life stages of galling aphids are in closed microenvironments^21,23^. Generally, hundreds to thousands of aphid individuals living in a gall are produced parthenogenetically by a single fundatrix^19,21,34^. If a fundatrix did not carry *Wolbachia* before she induces a gall, its offspring have basically no chance to be infected by *Wolbachia* in an enclosed environment. This means that *Wolbachia* can hardly spread across galling aphid populations. Moreover, galling aphids receive less pressure from natural enemies than non-galling aphids since they are protected by the gall wall^20,35^. Although vertical transfer is the predominant mode for *Wolbachia*, horizontal transfer also appeared in nature by infrequently. Horizontal transfer of *Wolbachia* was observed when infected and uninfected larvae of *Trichogramma* wasps shared the same hosts^31^. Also, Hymenopteran parasitoids of frugivorous *Drosophila* acquired *Wolbachia* through horizontal transmission with high frequency^36^. The three galling aphids in our study are host alternation, so they may readily acquire *Wolbachia* when they free-living on the secondary hosts by shared the same host mosses or attacked by parasitoid wasps which carried *Wolbachia*. All aphid samples of our study were collected from closed galls, so they are quite different in their living environments, natural enemy pressures and host plants compared to non-galling aphids^20,21,34^. This is probably the reason why they harbor *Wolbachia* strains or supergroups different to those in free-living aphids^3,4^.

Many marker genes have been used to detect *Wolbachia* in insects, but the consistency of these markers varies among different insect species^37–39^. The most conserved marker gene among different *Wolbachia* strains is the *16S rRNA* gene, which also provides more consistent PCR amplification. However, because the *16S rRNA* gene is highly conserved, non-target amplification occurs during PCR amplification^5^. The *wsp* gene evolves faster among different *Wolbachia* strains. Therefore, PCR amplification is not highly consistent with current primer pairs^7^. In this study, the primers for the *16S rRNA*, *gatB* and *fbpA* genes could detect *Wolbachia* in some galling aphid populations. However, primers for the *coxA*, *hcpA* and *ftsZ* genes detected *Wolbachia* only in *P. yunnanensis* (Table 1). Primers for the *wsp*, *groEL* and *gltA* genes did not detect *Wolbachia* in any samples, indicating that the primer regions in these genes varied and the primers could not achieve specific PCR amplifications. Our results also revealed that *Wolbachia* in the galling aphids was more difficult to detect using the existing primers possibly because of the variation in the sequences of target genes in the *Wolbachia* strains. Thus, the selection of more efficient and specific *Wolbachia* gene primers is needed to advance future research on *Wolbachia* analyses in aphids, especially in galling aphids.

## Materials and methods

### Aphid sample collection

Aphids of each population were collected from a mature gall. Fresh aphid galls were collected from host plants manually and dissected in the lab. Aphids were transferred to an Eppendorf tube from a gall, placed in 100% ethanol and then stored at − 20 °C until DNA extraction. Galls collected from each location within ten meters were treated as the same population. Since three generations of aphids are produced parthenogenetically by a mating female in a gall to achieve hundreds to thousands of offspring, aphids from a gall were treated as a clone. Galls collected from a location outside 10 m were treated as different populations.

Nine galling aphid species (Aphididae: Eriosomatinae: Fordini and Pemphigini) were collected from five host trees at eight locations in Yunnan, Sichuan, Hubei and Shaanxi provinces of China. *K. rhusicola* was the most common species and five populations were obtained from this species. *S. chinensis* was the second common species with three populations collected. The other six galling aphid species included *Floraphis meitanensis*, *Schlechtendalia peitan*, *Pemphigus yangcola*, *Pemphigus yunnanensis*, *Pemphigus populitransversus* and *Chaetogeoica folidentata*, each with only one population collected (Table 1).

### DNA extraction

Ten to twenty aphid individuals of a population were selected for DNA extraction. Genomic DNA was extracted using Dzup Genomic DNA Isolation Reagent (Sangon Biotech, China) from pooled aphids of the same clone following the manufacturer's protocol and was then stored at − 20 °C until detected.

### *Wolbachia* detection

DNA samples of 117 natural galling aphid populations were screened for the presence of *Wolbachia* strains. Detection was based on the amplification of the *16S rRNA* gene fragment (about 1,100 bp) with the *Wolbachia*-specific primers 16S 281F 5′-CTATAGCTGATCTGAGAGGAT-3′ and 16S 1372R 5′-YGCTTCGAGTGAAACCAATTC-3′ (Table 3)^4^. PCR procedures were an initial step of 94 °C for 3 min, followed by 35 cycles of 94 °C for 45 s, 55 °C for 60 s, and 72 °C for 90 s and a final step of 72 °C for 10 min. Amplified DNA products were electrophoresed on agarose gels and stained. PCR products were sequenced from both directions in an ABI 3730 DNA analyzer (Applied Biosystems, Foster City, CA, USA). Sequencing results were then checked by Blast in NCBI nr database (https://blast.ncbi.nlm.nih.gov/Blast.cgi). Only those samples, which were blasted to expected products of the specific primers, were selected for further analysis. These selected samples were further examined by PCR analyses of the genes *gltA*, *groEL*, *wsp*, *gatB*, *fbpA*, *coxA*, *hcpA* and *ftsZ* using specific primers based on previous reports (Table 3)^40–43^. PCR amplifications were carried out in 25 μl reaction volume, consisting of 1 μl DNA, 1 μl of forward and reverse primers (10 μmol/l), 12.5 μl Taq Master Mix (Sangon Biotech, China), and 9.5 μl ddH_2_O. PCR products (4 μl) were electrophoresed on a 1.5% agarose gel. Positive samples were further analyzed.

**Table 3:** Primer list used for *Wolbachia* detection.

Gene	Hypothetical product	Primer name and sequences (5′–3′)	Product size	Tm (°C)	References
*16S rRNA*	Ribosomal RNA 16S	16S 281F: CTATAGCTGATCTGAGAGGAT 16S 1372R: YGCTTCGAGTGAAACCAATTC	1,100	55	Wang et al.^4^
*16S rRNA*	Ribosomal RNA 16S	WspecF: CAT ACC TAT TCG AAG GGA TAG WspecR: AGC TTC GAG TGA AAC CAA TTC	440	52	Werren and Windsor^43^
*wsp*	Outer surface protein	*Wsp*81F: TGGTCCAATAAGTGATGAAGAAAC *Wsp*691R: AAAAATTAA ACGCTACTCCA	546	52	Zhou and Rousset^40^
*groEL*	Chaperonin GroEL	*GroEL*F: GGTGAGCAGTTRCARSAAGC *GroEL*R: TARCCRCGRTCAAAYTGCATRCCA	491	55	Wang et al.^4^
*ftsZ*	Cell division protein	*FtsZ*F: GTTGGYAAAGGTGCAGCAGAAGA *FtsZ*R: CGYACYCATTTKGCTGCAGMATCAA	524	53	Wang et al.^4^
*groEL*	Chaperonin GroEL	*groEL* F1: GGTGAGCAGTTGCAAGAAGC *groEL* R1: AGRTCTTCCATYTTRATTCC	491	55	Casiraghi et al*.*^41^
*gltA*	Citrate synthase	*glt*AF1: TACGATCCAGGGTTTGTTTCTAC *glt*AR1: CTCATTAGCTCCACCGTGTG	659	56	Casiraghi et al*.*^41^
*gatB*	Glutamyl-tRNA(Gln) amidotransferase, subunit B	*GatB* F1: GAKTTAAAYCGYGCAGGBGTT *GatB* R1: TGGYAAYTCRGGYAAAGATGA	497	54	Paraskevopoulos et al.^42^
*coxA*	Cytochrome coxidase, subunit I	*CoxA*-F1: TTGGRGCRATYAACTTTATAG *CoxA*-R1: CTAAAGACTTTKACRCCAGT	488	55	Paraskevopoulos et al.^42^
*hcpA*	Conserved hypothetical protein	*HcpA*-F1: GAAATARCAGTTGCTGCAAA *HcpA*-R1: GAAAGTYRAGCAAGYTCTG	515	53	Paraskevopoulos et al.^42^
*fbpA*	Fructose-bisphosphate aldolase	*FbpA*-F1: GCTGCTCCRCTTGGYWTGAT *FbpA*-R1: CCRCCAGARAAAAYYACTATTC	509	59	Paraskevopoulos et al.^42^

### Sequencing

Nine genes *16S rRNA*, *gltA*, *groEL*, *wsp*, *gatB*, *fbpA*, *coxA*, *hcpA* and *ftsZ* of *Wolbachia* were amplified and sequenced from the populations harboring *Wolbachia*. PCR products were sequenced from both directions in an ABI 3730 DNA analyzer. Original sequences were manually processed and then assembled by DNA Star Lasergene v7.1-SeqMan.

### Nucleotide sequence accession numbers

Twelve gene sequences of *16S rRNA*, *gltB*, *fbpA*, *coxA*, *hcpA* and *ftsZ* generated in this study have been deposited in the GenBank database under accession numbers MT554837-MT554842 and MT634226-MT634228.

### Sequence alignment and phylogenetic analysis

Genes representative of *Wolbachia* strains from different supergroups were selected from the NCBI database (https://www.ncbi.nlm.nih.gov), and used to classify *Wolbachia* strains detected in our aphid samples. Sequence alignments were carried out using ClustalX 1.83^44^. Maximum likelihood (ML) and Bayesian inference (BI) were used for phylogenetic analysis. The ML analysis was carried out with MEGA 6.0 and node support rates were evaluated with 1,000 bootstrap replicates. The Bayesian inference (BI) method was performed in MrBayes 3.1.2^45^. In MrBayes, four chains (one cold and three heated chains) which were run for 4 million generations. Trees were sampled every 100 generations, and the first 25% of samples were discarded as burn-in. From the remaining trees, 50% majority-rule consensus trees were generated. Posterior probabilities were computed from the remaining trees. To obtain the appropriate evolution model, the parameters were evaluated using the Akaike Information Standard (AIC) in MrModeltest 2.3^46^. Using this method, the following gene models were obtained: the HKY + G model of *16S rRNA* gene, the GTR + G model of *ftsZ* gene, the HKY + G model of *coxA* gene, the GTR + I + G model of *gatB* gene and the HKY + G model of *fbpA* gene.

## References

[1] Werren JH (1997). Biology of *Wolbachia*. Annu. Rev. Entomol..

[2] Stouthamer R, Breeuwer JA, Hurst GD (1999). *Wolbachia pipientis*: microbial manipulator of arthropod reproduction. Annu. Rev. Microbiol..

[3] Augustinos AA (2011). Detection and characterization of *Wolbachia* infections in natural populations of aphids is the hidden diversity fully unraveled. PLoS ONE.

[4] Wang Z, Su XM, Wen J, Jiang LY, Qiao GX (2014). Widespread infection and diverse infection patterns of *Wolbachia* in Chinese aphids. Insect Sci..

[5] Bing XL (2014). Diversity and evolution of the *Wolbachia* endosymbionts of *Bemisia* (Hemiptera: Aleyrodidae) whiteflies. Ecol. Evol..

[6] Glowska E, Dragun-Damian A, Dabert M, Gerth M (2015). New *Wolbachia* supergroups detected in quill mites (Acari: Syringophilidae). Infect. Genet. Evol..

[7] Baldo L (2006). Multilocus sequence typing system for the endosymbiont *Wolbachia pipientis*. Appl. Environ. Microbiol..

[8] Rigaud T (1991). Feminizing endocytobiosis in the terrestrial crustacean *Armadillidium**vulgare* Latr. (Isopoda): recent acquisitions. Endocytobiosis Cell Res..

[9] Gdd H, Men M, Walker LE (1993). The importance of cytoplasmic male killing elements in natural populations of the two spot ladybird, *Adalia bipunctata* (Linnaeus) (Coleoptera: Coccinellidae). Biol. J. Linn. Soc..

[10] Stouthamer R, Breeuwer JAJ, Luck RF, Werren JH (1993). Molecular identification of microorganisms associated with parthenogenesis. Nature.

[11] Werren JH, Baldo L, Clark ME (2008). *Wolbachia*: master manipulators of invertebrate biology. Nat. Rev. Microbiol..

[12] Hedges LM, Brownlie JC, O’Neill SL, Johnson KN (2008). *Wolbachia* and virus protection in insects. Science.

[13] Moreira LA (2009). *Wolbachia* symbiont in *Aedes aegypti* limits infection with dengue, Chikungunya, and Plasmodium. Cell.

[14] Mcmeniman CJ (2009). Stable introduction of a life-shortening *Wolbachia* infection into the mosquito *Aedes aegypti*. Science.

[15] Hosokawa T, Koga R, Kikuchi Y, Meng XY, Fukatsu T (2010). *Wolbachia* as a bacteriocyte-associated nutritional mutualist. Proc. Natl. Acad. Sci..

[16] Fast EM (2011). *Wolbachia* enhances *Drosophila* stem cell proliferation and target the germline stem cell niche. Science.

[17] Bourtzis K (2008). *Wolbachia*-based technologies for insect pest population control. Adv. Exp. Med. Biol..

[18] Blackman RL, Eastop VF (2006). Aphids on the World’s herbaceous plants and shrubs.

[19] Zhang GX, Qiao GX, Zhong TS, Zhang WY (1999). Fauna Sinica Insecta 14, Homoptera, Mindaridae and Pemphigidae.

[20] Wool D (2004). Galling aphids: specialization, biological complexity, and variation. Annu. Rev. Entomol..

[21] Stone GN, Schonrogge K (2003). The adaptive significance of insect gall morphology. Trends Ecol. Evol..

[22] Schultz JC, Edger PP, Body MJA, Appel HM (2019). A galling insect activates plant reproductive programs during gall development. Sci. Rep..

[23] Moran NA (1992). The evolution of aphid life cycles. Annu. Rev. Entomol..

[24] Zhang GX, Zhong TS (1983). Economic insect fauna of China. 25 Homoptera: Aphidinea.

[25] Yang ZX (2011). High yield cultivation techniques of Chinese Gallnut.

[26] Jeyaprakash A, Hoy MA (2000). Long PCR improves *Wolbachia* DNA amplification: *wsp* sequences found in 76% of sixty-three arthropod species. Insect Mol. Biol..

[27] Tsuchida T, Koga R, Shibao H, Matsumoto T, Fukatsu T (2002). Diversity and geographic distribution of secondary endosymbiotic bacteria in natural populations of the pea aphid, *Acyrthosiphon pisum*. Mol. Ecol..

[28] Gomez-Valero L (2004). Coexistence of *Wolbachia* with *Buchnera aphidicola* and a secondary symbiont in the aphid *Cinara cedri*. J. Bacteriol..

[29] Wang Z, Shen ZR, Song Y, Liu HY, Li ZX (2009). Distribution and diversity of *Wolbachia* in different populations of the wheat aphid *Sitobion miscanthi* (Hemiptera: Aphididae) in China. Eur. J. Entomol..

[30] Reuter M, Keller L (2003). High levels of multiple *Wolbachia* infection and recombination in the ant *Formica exsecta*. Mol. Biol. Evol..

[31] Huigens ME (2004). Natural interspecific and intraspecific horizontal transfer of parthenogenesis-inducing *Wolbachia* in *Trichogramma* wasps. Proc. R. Soc. Lond. B..

[32] Guo J (2017). Nine facultative endosymbionts in aphids. A review. J. Asia-Pac. Entomol..

[33] De Clerck C (2015). A metagenomic approach from aphid’s hemolymph sheds light on the potential roles of co-existing endosymbionts. Microbiome.

[34] Shao SX, Yang ZX, Chen XM (2012). Gall development and clone dynamics of the galling aphid *Schlechtendalia chinensis* (Hemiptera: Pemphigidae). J. Econ. Entomol..

[35] Oliver KM (2010). Facultative symbionts in aphids and the horizontal transfer of ecologically important traits. Annu. Rev. Entomol..

[36] Vavre F, Fleury F, Lepetit D, Fouillet P, Boulétreau M (1999). Phylogenetic evidence for horizontal transmission of *Wolbachia* in host-parasitoid associations. Mol. Biol. Evol..

[37] Gerth M (2016). Classification of *Wolbachia* (Alphaproteobacteria, Rickettsiales): No evidence for a distinct supergroup in cave spiders. Infect. Genet. Evol..

[38] Lefoulon E (2016). Breakdown of coevolution between symbiotic bacteria *Wolbachia* and their filarial hosts. PeerJ.

[39] Bleidorn C, Gerth M (2018). A critical re-evaluation of multilocus sequence typing (MLST) efforts in *Wolbachia*. FEMS Microbiol. Ecol..

[40] Zhou W, Rousset F, O'Neill S (1998). Phylogeny and PCR-based classification of *Wolbachia* strains using *wsp* gene sequences. Proc. R. Soc. Lond. B..

[41] Casiraghi M (2005). Phylogeny of *Wolbachia pipientis* based on *gltA*, *groEL* and *ftsZ* gene sequences: clustering of arthropod and nematode symbionts in the F supergroup, and evidence for further diversity in the *Wolbachia* tree. Microbiology.

[42] Paraskevopoulos C, Bordenstein SR, Wernegreen JJ, Werren JH, Bourtzis K (2006). Toward a *Wolbachia* multilocus sequence typing system: discrimination of *Wolbachia* strains present in *Drosophila* species. Curr. Microbiol..

[43] Werren JH, Windsor DM (2000). Wolbachia infection frequencies in insects: evidence of a global equilibrium?. Proc. Biol. Sci..

[44] Thompson JD, Gibson TJ, Plewniak F, Jeanmougin F, Higgins DG (1997). The CLUSTAL_X windows interface: flexible strategies for multiple sequence alignment aided by quality analysis tools. Nucleic Acids Res..

[45] Ronquist F, Huelsenbeck JP (2003). MRBAYES 3: Bayesian phylogenetic inference under mixed models. Bioinformatics.

[46] Nylander JAA (2004). MrModeltest v2.2. Program distributed by the author.

